# Sensitivity of Intra- and Intermolecular Interactions of Benzo[h]quinoline from Car–Parrinello Molecular Dynamics and Electronic Structure Inspection

**DOI:** 10.3390/ijms22105220

**Published:** 2021-05-14

**Authors:** Jarosław J. Panek, Joanna Zasada, Bartłomiej M. Szyja, Beata Kizior, Aneta Jezierska

**Affiliations:** 1 Faculty of Chemistry, University of Wrocław, ul. F. Joliot-Curie 14, 50-383 Wrocław, Poland; jaroslaw.panek@chem.uni.wroc.pl; 2 Department of Fuels Chemistry and Technology, Wrocław University of Science and Technology, ul. Gdańska 7/9, 50-344 Wrocław, Poland; joanna.zasada@pwr.edu.pl (J.Z.); b.m.szyja@pwr.edu.pl (B.M.S.); beata.kizior@pwr.edu.pl (B.K.)

**Keywords:** benzo[h]quinoline, gas phase, solvent, crystalline phase, CPMD, O-H stretching envelope, 1D and 2D Pmf, CDFT, ELF

## Abstract

The O-H...N and O-H...O hydrogen bonds were investigated in 10-hydroxybenzo[h]quinoline (HBQ) and benzo[h]quinoline-2-methylresorcinol complex in vacuo, solvent and crystalline phases. The chosen systems contain analogous donor and acceptor moieties but differently coupled (intra- versus intermolecularly). Car–Parrinello molecular dynamics (CPMD) was employed to shed light onto principle components of interactions responsible for the self-assembly. It was applied to study the dynamics of the hydrogen bonds and vibrational features as well as to provide initial geometries for incorporation of quantum effects and electronic structure studies. The vibrational features were revealed using Fourier transformation of the autocorrelation function of atomic velocity and by inclusion of nuclear quantum effects on the O-H stretching solving vibrational Schrödinger equation a posteriori. The potential of mean force (Pmf) was computed for the whole trajectory to derive the probability density distribution and for the O-H stretching mode from the proton vibrational eigenfunctions and eigenvalues incorporating statistical sampling and nuclear quantum effects. The electronic structure changes of the benzo[h]quinoline-2-methylresorcinol dimer and trimers were studied based on Constrained Density Functional Theory (CDFT) whereas the Electron Localization Function (ELF) method was applied for all systems. It was found that the bridged proton is localized on the donor side in both investigated systems in vacuo. The crystalline phase simulations indicated bridged proton-sharing and transfer events in HBQ. These effects are even more pronounced when nuclear quantization is taken into account, and the quantized Pmf allows the proton to sample the acceptor area more efficiently. The CDFT indicated the charge depletion at the bridged proton for the analyzed dimer and trimers in solvent. The ELF analysis showed the presence of the isolated proton (a signature of the strongest hydrogen bonds) only in some parts of the HBQ crystal simulation. The collected data underline the importance of the intramolecular coupling between the donor and acceptor moieties.

## 1. Introduction

Qualitative and quantitative prediction of intra- and intermolecular forces responsible for the self-assembly of molecules has been widely investigated due to its importance in the design of new molecules with desired properties [1,2]. Among the various non-covalent forces that need to be considered, one should name—in a rough series from the strongest to the weakest ones—the electrostatic attraction between charged moieties (which are not applicable to our current study), hydrogen bonds (HBs) providing a wide range of interaction energies, halogen, chalcogen, tetrel or pnicogen bonds—objects of recent intensive research [3,4,5], non-classical forms of hydrogen bonding (e.g., C-H...π contacts or charge-inverted HBs) [6,7], and omnipresent yet the weakest of all, dispersion forces. Most of these forces are very sensitive to the modifications of the molecular skeleton, influence of the inductive and steric effects, or the changes in the environment (solvent or crystal fields). This makes the intermolecular forces such significant components in the understanding of the self-assembly processes or in the design of the molecular materials. The aforementioned sensitivity is also related to such facts that generally the role of intramolecular contacts in the molecule differs from the intermolecular forces, or that many weaker contacts can potentially outweigh a strong, but lone hydrogen bond.

**Figure 1:**
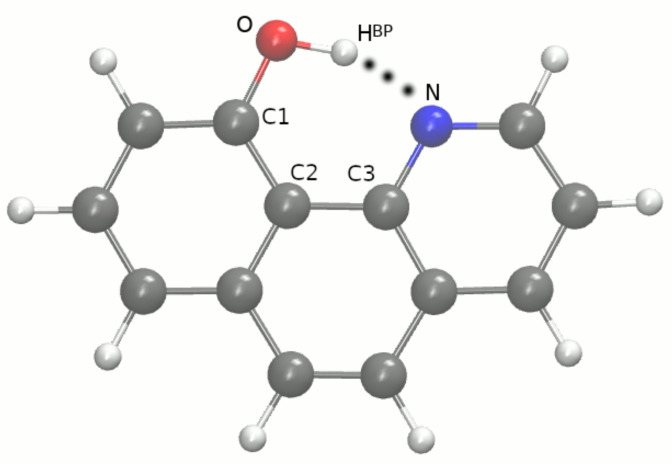
10-hydroxybenzo[h]quinoline (HBQ) model for gas-phase CPMD simulation. Only atoms involved in the intramolecular hydrogen bond and quasi-ring formation are indicated. The atoms notation was prepared for the study. The dotted line indicates the presence of an intramolecular hydrogen bond. Atom coloring scheme: grey—carbon atoms, red—oxygen atom, blue—nitrogen atom, white—hydrogen atom.

**Figure 2:**
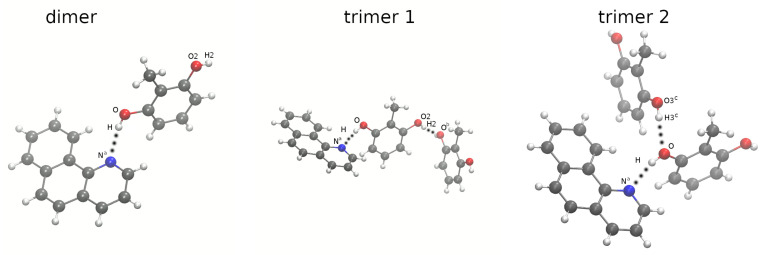
Model of dimer and trimers of benzo[h]quinoline-2-methylresorcinol for gas-phase CPMD simulations with atoms involved in the intermolecular hydrogen bonds formation. The atoms notation was prepared for the study and only atoms of interest are marked. Dotted lines indicate the presence of the intermolecular hydrogen bonds. Atom coloring scheme: grey—carbon atoms, red—oxygen atom, blue—nitrogen atom, white—hydrogen atom. The trimers are built from the crystal structure [9] taking the central 2-methylresorcinol as a reference and then generating the surrounding molecules using the symmetry codes ^*a*^(x,y,z) for HBQ, ^*b*^($\frac{1}{2}$+x,$\frac{1}{2}-$y,$\frac{1}{2}$+z) for the trimer 1, and ^*c*^($-\frac{1}{2}$+x,$\frac{1}{2}-$y,$-\frac{1}{2}$+z) for the O3${}^{c}$-H3${}^{c}$ molecule of the trimer 2.

Therefore, we focused on two quinoline containing systems: 10-hydroxybenzo[h]quinoline (HBQ) [8] and benzo[h]quinoline-2-methylresorcinol complex [9], see Figure 1 and Figure 2 respectively. The HBQ has found an important practical application as a reagent in the preparation of optical filter agents in photographic emulsion [10]. However, the most spectacular and important is the Excited-State Intramolecular Proton Transfer (ESIPT), which the compound exhibits. Many studies have been devoted to the detailed description of the process, see e.g., Refs. [11,12,13,14,15,16,17,18]. In the electronic ground state S${}_{0}$, the molecule exists in the enol form with the bridged proton attached to the oxygen (proton-donor atom). The excitation process of the molecule to the S${}_{1}$ state is associated with the proton transfer to the nitrogen atom. The process is barrier-less and the keto form of the molecule is formed. A reverse proton transfer occurs (also barrier-less) after the radiative deactivation to the electronic ground state (the initial enol form is obtained). On the basis of the compound, new derivatives with photophysical properties were developed [19,20,21,22]. In general, molecules that display the ESIPT accompanied by a large Stokes shift could be used as e.g., photoacids [23], UV photostabilizers [24], fluorescent solar concentrators [25], laser dyes [26] or organic light-emitting devices [27]. The second investigated system is a product of cocrystallization experiments of 2-methylresorcinol with several N-bases [9]. The experiments were performed to identify a selected and favorable crystalization routes in relevant structural conditions. The targeting synthon-based crystallization was enhanced by chosen conformers combination to obtain stoichiometric ternary solids. At this point, it is necessary to clarify that ternary cocrystals are molecular solids containing three discrete solid organic compounds in their basic unit cell [28]. The applied procedure is equivalent to the combinatorial synthesis of crystals.

The two chosen systems for the study contain intra- and intermolecular hydrogen bonds. The presence of hydrogen bonds plays an important role in the structure stabilization, molecular association processes as well as it influences the binding energy and spectroscopic properties [29]. The hydrogen bonds in these systems are as similar to possible: the donor is a phenolic -OH group, while the acceptor is a nitrogen heteroatom embedded in a benzo[h]quinoline skeleton. The most important difference is that the HBQ molecule contains an intramolecular hydrogen bond, while the benzo[h]quinoline-2-methylresorcinol complex is formed via an intermolecular interaction. This allows us to analyze static as well as time-evolving properties of two similarly composed, but fundamentally diverse hydrogen bridges. What properties will be analyzed, and what are our expectations as to the potential differences between the intra- and intermolecular cases?

An initial insight into the behavior of HBQ as well as benzo[h]quinoline-2-methylresorcinol complex, with emphasis on the molecular forces governing the behavior of these two systems, was carried out based on the DFT static models [30]. The most important findings of this study amounted to the following facts: the influence of the polar environment (crystal field) on aromaticity was rather minor; while the spontaneous proton transfer was not preferable in the electronic ground state of both HBQ and benzo[h]quinoline-2-methylresorcinol complex, it was possible in the excited state as modelled by the TD-DFT approach; and finally—most important from the point of view of intermolecular non-covalent interactions—the Symmetry-Adapted Perturbation Theory (SAPT) analysis showed the O-H...N intermolecular hydrogen bond of the benzo[h]quinoline-2-methylresorcinol complex to be only ca. twice stronger than the C-H...O and C-H...π contacts present in the dimers taken from the crystal of 10-hydroxybenzo[h]quinoline. This shows that more diverse approach to these two systems would be beneficial, possibly including dynamical effects. Especially the dynamical nature of the hydrogen bond, the dominant binding factor, should be taken care of.

The hydrogen bond formation and its influence on the infrared (IR) spectra have been commonly accepted as the most important feature of the interaction. It concerns mainly the absorption band of the D-H stretching group (where D is the proton donor). In the IR spectra, a strong frequencies shift towards lower wavenumbers is observed for the D-H group. The integral intensity and the half-width of the band are multiple increased [31,32,33]. In the current study, the metric and vibrational features of the studied systems were investigated based on Car–Parrinello molecular dynamics (CPMD) [34]. Additionally, the quantum corrections to the O–H stretching have been added by solving the nuclear Schrödinger equation for the trajectory snapshots of the O–H motion. The CPMD methods are classical in the sense of nuclear motion treatment. In order to reproduce correctly, some phenomena observed at the molecular level a quantum effects inclusion is necessary [35,36]. The quantization of the O-H motion enables a more accurate theoretical description qualitatively closer to the experimental data. The electronic structure was analyzed using Constrained Density Functional Theory (CDFT) [37] and Electron Localization Function (ELF) [38].

We paid special attention to the intra- and intermolecular hydrogen bonds involving benzo[h]quinoline molecule. Our study extends previous results of many researchers, especially the cocrystallization experimental study leading to the benzo[h]quinoline-2-methylresorcinol complex [9], which show indeed the primary role of hydrogen bonds in the formation of long-range ordered structures for benzo[h]quinolines. In case of the two-component complex, the formed cocrystal exhibits diverse hydrogen bonding schemes, i.e., an O-H...N contact and two types of O-H...O bridges, which prompted us to include this system in our analysis. In particular, we investigated: (i) the bridged proton mobility in the gas and crystalline phases; (ii) the spectroscopic properties using two approaches: the Fourier transformation of the time autocorrelation function of the atomic velocity [39] and the O-H stretching envelope method (where the quantum effects were incorporated a posteriori [31]; (iii) free-energy profiles by the potential of mean force (Pmf) computed using CPMD trajectory and with inclusion of quantum effects a posteriori; (iv) the electron density changes in the proton reaction path using Constrained Density Functional Theory (CDFT) [37] for the intermolecular interactions; (v) the decomposition of the molecular space using Electron Localization Function (ELF) theory to estimate the hydrogen bonds strength [38].

The main aim of the study was the development of interaction models covering various factors responsible for the structure arrangement and molecular self-assembly. Up to our knowledge this is the first CPMD study of HBQ and benzo[h]quinoline-2-methylresorcinol. It covers a wide range of molecular interactions with an emphasis on incorporation of quantum effects and electronic structure description given by CDFT and ELF approaches. The special feature of this study is the comparison of two systems with very similar donor and acceptor moieties, but with totally different coupling between them. The understanding of two such systems could be useful in the design of new benzo[h]quinolines with desired photochemical/photophysical properties. In particular, enriched understanding of the interplay between different types of hydrogen bonding and other, weaker intermolecular forces, potentially leading to preferential formation of diverse self-assembled structures, can be used for rational design of composite systems such as cocrystals [9].

## 2. Results and Discussion

### 2.1. Car–Parrinello Molecular Dynamics in the Gas and Crystalline Phases—Structural and Vibrational Aspects

Car–Parrinello molecular dynamics (CPMD) simulations were performed for the HBQ molecule (see Figure 1) and dimer and trimers formed by benzo[h]quinoline-2-methylresorcinol (see Figure 2) in vacuo. The models for the crystalline phase CPMD runs are presented in Figures S1 and S2 respectively and they were prepared based on experimental data available [8,9].

A detailed discussion of selected metric parameters of all studied systems is presented in the Supporting Information. The HBQ molecule possesses a strong, resonance-assisted hydrogen bond (RAHB), [40] see Table S1. The presence of an intramolecular hydrogen bond resulted in the formation of a quasi-ring, which additionally stabilizes the structure. Therefore, the discussion of the quasi-ring remaining metric parameters time evolution was added beside the intramolecular hydrogen bond.

**Figure 3:**
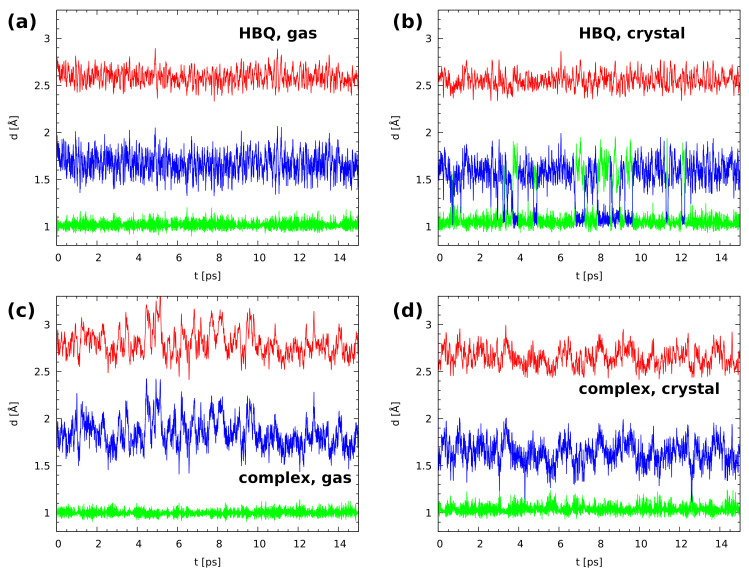
Time evolution of the metric parameters of the O-H...N hydrogen bridges in the investigated systems. (**a**): HBQ in the gas phase, (**b**): HBQ in the crystal, (**c**): the benzo[h]quinoline-2-methylresorcinol complex in the gas phase, (**d**): the complex in the crystal. Color coding of the graphs: red—d(O...N), green—r(O-H), blue: r(H...N).

In Figure 3 (upper part) the time evolution of geometric parameters (bond lengths) of the O-H^BP^...N hydrogen bridge of HBQ molecule is presented. The gas-phase data indicated that the bridged proton is located on the donor side. Even proton-sharing events were not noticed. The proton is labile, but in the ground electronic state, it is not able to enter the acceptor-atom domain during the 15 ps run. The CPMD crystalline phase simulation results showed a strong mobility of the bridged proton with frequent proton-transfer phenomena and proton-sharing events. According to our knowledge this is the first study of the HBQ solid-state property investigations based on CPMD approach. The bridged proton was moving to the acceptor-atom side, stayed there for a short period of time (on the order of 0.5 ps), and kept returning to the donor-atom side. The proton resided on the acceptor side for longer and shorter periods of time as shown in Figure 3. Comparing our findings for the gas phase and solid-state CPMD simulations, the environmental effects play a significant role, because they were able to change the bridged proton dynamics introducing events not observed for the isolated molecule. However, the enol form of the HBQ is preferable as well in the crystalline phase.

In the case of benzo[h]quinoline-2-methylresorcinol complex and cocrystal, the intermolecular hydrogen bonds of the O-H...N and O-H...O type were analyzed, see Figure 2 and Table S2. The intermolecular interactions could be considered to be strong (O-H...N) and middle-strong (O-H...O). A detailed analysis of the selected metric parameters is given in the Supporting Information.

Time-evolution results of intermolecular hydrogen bonds of benzo[h]quinoline-2-methylresorcinol complex are presented in Figure 3, bottom part. It forms a network of middle-strength intermolecular hydrogen bonds. The gas-phase data showed a very strong dynamics of the O...N${}^{a}$ interatomic distance. A very similar tendency was noticed for the intermolecular hydrogen bond H...N${}^{a}$. There were neither noticeable proton-sharing events nor proton-transfer phenomena. However, a correlation between the O..N${}^{a}$ and H...N${}^{a}$ dynamics is visible, see Figure 3. Summarizing, the bridged proton is located on the donor side during the 15 ps of the CPMD run. In the crystalline phase simulation, there was noticed also strong flexibility of the hydrogen bridge. As it is shown in Figure 3 (panel (d)), the O...N${}^{a}$ interatomic distance was strongly changing during the CPMD run. A strong flexibility of the H...N${}^{a}$ hydrogen bond was noticed as well. Moreover, it is worth underlining that a few contacts were observed with the proton in the middle between the donor and acceptor atoms. Such events were observed after ca. 3.0, 4.2, and 12.5 ps of the CPMD simulation. The presence of the proton-sharing events in the crystalline phase indicates the increased strength of the hydrogen bond with respect to the isolated system. Therefore, the environmental effect, e.g., neighboring molecules, intermolecular interactions as well as crystal fields can affect the hydrogen bonding properties. In Figure S3 there is presented time evolution of O2-H2...O${}^{b}$ and O3${}^{c}$-H3${}^{c}$...O intermolecular hydrogen bridges in the crystalline phase. The dynamics of both hydrogen bonds are very similar. Strong changes were observed for O2...O${}^{b}$ and O3${}^{c}$...O interatomic distances as well as for H2...O${}^{b}$ and H3${}^{c}$...O hydrogen bonds during the CPMD run. However, there were not noticed proton-sharing events and the bridged hydrogen was attached to the donor atom. Therefore, we can conclude that the O-H...O hydrogen bonds are weaker comparing to the O-H...N intermolecular interaction.

The spectroscopic properties analysis was carried out based on atomic velocity power spectra. This method allows for easy separation of atomic contributions, which was used in the current study with particular attention paid to the bridged proton dynamics. Experimental vibrational spectra can suffer from the overlap of modes, especially for the broad absorption regions of stretching vibrations involving the bridged protons. An analysis of the atomic velocity power spectra does not suffer from this phenomenon, but there are two important issues to be remembered. First, this is an analysis of a classical Newtonian trajectory of nuclei, therefore mechanical couplings between the oscillators are allowed in a different way than in the true, quantum dynamics (i.e., we cannot essentially see tunneling splitting, overtones, Fermi resonances, Evans holes, etc.). Second, the intensities are arbitrary and linked rather to the mechanical amplitudes, not to the observable intensities associated with an electric dipole moment. The second of these issues can be neglected for the type of discussion we will carry out, while the first one will be partially dealt with in the next section on the a posteriori inclusion of quantum effects.

**Figure 4:**
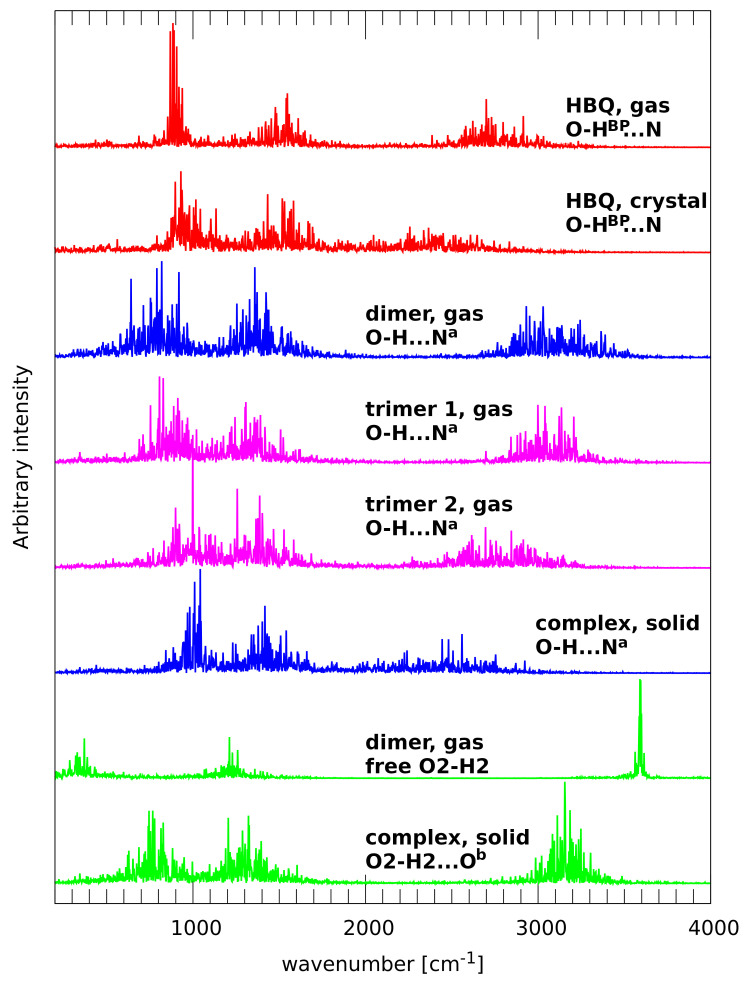
Vibrational signatures of the hydroxyl protons (hydrogen bridge protons with exception of the gas-phase heterodimeric complex of benzo[h]quinoline with 2-methylresorcinol, in which one of the -OH groups is free).

The atomic velocity power spectra of atomic motions of the bridged hydrogen atoms are gathered in Figure 4. The first visible feature of these charts is a striking difference between the bridges with nitrogen vs. oxygen atoms as proton acceptors. Even the division between the intra- and intermolecular bridges seems less important. We will start the discussion with the case of HBQ, containing the strongest hydrogen bond among the analyzed systems. The relation of the IR spectrum of HBQ to its internal dynamics was recently discussed based on broadband infrared pump-probe spectroscopy [41]. This experimental study reports the center of the $\nu_{OH}$ band of HBQ in CCl${}_{4}$ to be located at 2771 cm${}^{-1}$. Our results are in perfect agreement: the high-wavenumber part of the atomic velocity power spectrum for the gas-phase CPMD simulation of HBQ extends from ca. 2400 to 3250 cm${}^{-1}$, peaks at 2700 cm${}^{-1}$ and is centered at 2800 cm${}^{-1}$. Several factors combine to provide such an agreement: first, the experimental measurements were taken in CCl${}_{4}$ [41], which is a low-polarity solvent, but still its influence leads to red shift in the $\nu_{OH}$ bands. Second, the nature of CPMD leads to the effect of slowing down the nuclear dynamics by (hopefully weak) coupling to the fictitious orbital dynamics. This effect, in addition to the soft DFT potential energy surface, was found to yield wavenumbers lower by even 50 cm${}^{-1}$ then the experiment in CPMD calculations of aqueous solutions [42]. This discussion can be extended to the remaining part of the vibrational analysis.

The case of HBQ in the solid state shows how easy to modify are the vibrational features of strong hydrogen bonds. We have already discussed how the presence of a crystal field modifies the proton bridge dynamics and allows for frequent proton-sharing and transfer phenomena. This results in a strong anharmonicity and time modulation of the potential energy surface, and this leads to a significant shift of the $\nu_{OH}$ band to lower wavenumbers, extending from as low as 1800 cm${}^{-1}$ up to 2900 cm${}^{-1}$. At the lower wavenumber range, this makes the $\nu_{OH}$ band overlap with the contributions of bending modes and couplings with heavy atom motions. The amplitudes and time scales of the modulation of the proton dynamics are however not sufficient to develop separate regimes of proton motion. We have observed such phenomenon in the CPMD studies on the hydrogen bridge dynamics in “proton sponges”: heavy tetramethylguanidine substituents of 1,8-bis(tetramethylguanidino)naphthalene enforced periods of very anharmonic, low-wavenumber proton motion interspersed with “almost free N-H” high-wavenumber dynamics [43]. Another case of diversity of proton dynamics and its manifestation in the vibrational spectrum was reported for nitranilic acid hexahydrate, a system containing highly anharmonic Zundel cation [44]. The dynamical changes of the O-H distances led to the diversity of wavenumber red shifts and half-widths of the O-H stretching bands, leading to a broad, complex absorption of the protonic band of the Zundel cation.

The benzo[h]quinoline-2-methylresorcinol complex exhibits intermolecular O-H...N bridge, present in the gas-phase dimer as well as in the cocrystal (where the nitrogen atom is denoted as N${}^{a}$ to reflect the fact of its being generated by symmetry operation). The vibrational signature of the $\nu_{OH}$ band in the gas phase is shifted towards higher wavenumbers (2700 to 3500 cm${}^{-1}$) in comparison to the intramolecular bridge of HBQ however, this distinction vanishes in the solid state. The $\nu_{OH}$ signature of the O-H...N${}^{a}$ bridge, ending at 1900 cm${}^{-1}$, practically blends with the medium-wavenumber features starting from ca. 1750 cm${}^{-1}$. This fact indicates that the middle-strong O-H...N intermolecular hydrogen bridge of the gas-phase dimer is significantly strengthened in the solid state. It is worthwhile to observe in Figure 4 the gradual progression of this strengthening, starting from the dimer, through trimer 1, trimer 2, to the cocrystal. As stated above, the $\nu_{OH}$ mode of the O-H...N${}^{a}$ bridge spans the range 2700–3500 cm${}^{-1}$ for the dimer. This bridge in trimer 1 is not directly modulated, but the effects of the formation of the O2-H2...O${}^{b}$ bridge is reflected in the shift of the $\nu_{OH}$ upper range down to 3400 cm${}^{-1}$. On the other hand, trimer 2 contains O3${}^{c}$-H3${}^{c}$...O bridge directly affecting the oxygen atom of the O-H...N${}^{a}$ bridge. The span of the $\nu_{OH}$ mode is in this case 2300–3300 cm${}^{-1}$, which is, however, still far from the fully polarized bridge of the HBQ in the cocrystal (1900–2900 cm${}^{-1}$).

The studied two-component trimers and cocrystal possess also bridges of the O-H...O type, formed with neighboring molecules (see Supporting Information for details). These bridges, not present in the dimer and differently organized in the trimers (see Figure 2), are symmetry-equivalent in the crystal. The signature of the O2-H2 stretching mode in the dimer corresponds to the free O-H case: a sharp, narrow band centered at 3600 cm${}^{-1}$. In the crystal, this band is centered at 3150 cm${}^{-1}$ and ranges from 2900 to 3400 cm${}^{-1}$. This is a significant shift but still corresponds only to middle-strong hydrogen bonds.

### 2.2. A Posteriori Inclusion of Quantum Effects to the O-H Stretching and Potential of Mean Force (Pmf)

**Figure 5:**
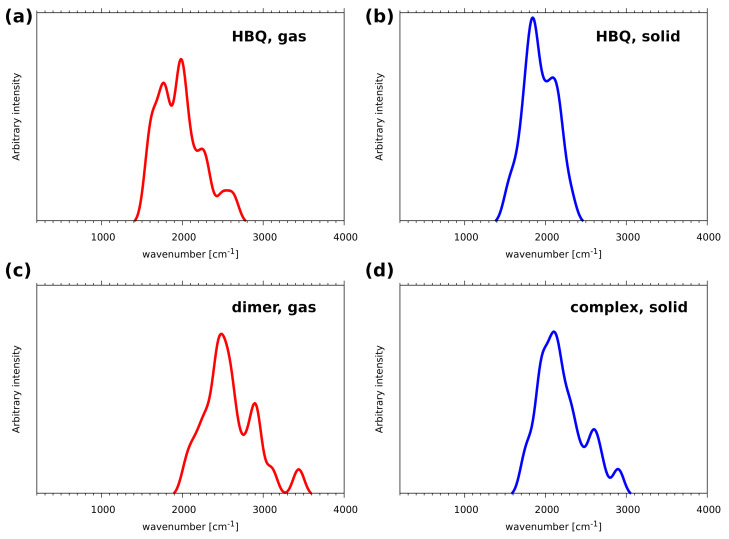
The O-H stretching mode envelopes calculated by a posteriori quantum corrections to the proton motion in the investigated O-H...N hydrogen bridges (**a**): HBQ in the gas phase, (**b**): HBQ in the crystal, (**c**): the benzo[h]quinoline-2-methylresorcinol complex in the gas phase, (**d**): the complex in the crystal).

The inclusion of quantum effects to the O-H proton motion, carried out using a posteriori “snapshot-envelope” technique, [31] resulted in the envelopes of the $\nu_{OH}$ mode presented in Figure 5. The most important feature of these quantum-corrected envelopes is that the $\nu_{OH}$ signatures are shifted towards lower wavenumbers than their classical counterparts estimated based on atomic velocity power spectra (see Figure 4). For example, the quantized O-H^BP^ stretching band of HBQ in the gas phase does not extend above 2800 cm^−1^, while the classical approximation yields the upper limit of this band at ca. 3250 cm^−1^. Interestingly, the lower limit of this band in HBQ, close to 1500 cm^−1^, is almost identical for the gas phase and solid state. The proton in the intermolecular hydrogen bond of the benzo[h]quinoline-2-methylresorcinol complex experiences distance variations of larger amplitude and it is shifted towards longer distances—this makes the $\nu_{OH}$ signature of the complex extending to much higher wavenumbers. It seems however that the combination of the DFT functional and quantum effects provides band positions shifted towards lower wavenumbers in comparison with the experiment.

**Figure 6:**
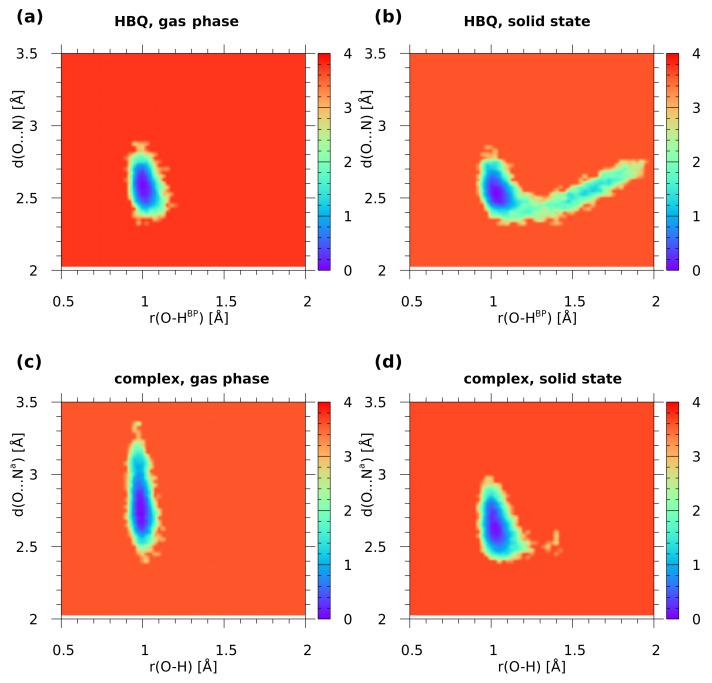
Results of the CPMD simulation: the potential of mean force (Pmf) for proton motion in the investigated O-H...N hydrogen bridges (**a**): HBQ in the gas phase, (**b**): HBQ in the crystal, (**c**): the benzo[h]quinoline-2-methylresorcinol complex in the gas phase, (**d**): the complex in the crystal). Color scale—Pmf in kcal/mol.

The potential of mean force (Pmf) was computed with classical atomic nuclei treatment as well as with the inclusion of quantum effects. The calculations were performed using gas phase and solid-state CPMD trajectories. One- and two-dimensional (1D and 2D) Pmf results for proton motion of the investigated O-H...N hydrogen bonds are presented in Figure S4 and Figure 6. In the gas phase for HBQ molecule (upper graph, left panel) only one free-energy minimum was detected with O-H${}^{BP}$ equal ca. 1.0 Å. This agrees with the time evolution of metric parameters study where it was found that the bridged proton resides solely on the donor side during the CPMD run. In the solid state a radically different picture was found (upper graph, right panel). Two minima appeared: the deeper one located at the proton-donor side and the shallow one at the proton-acceptor side. They are separated by a small barrier of ca. 2.5 kcal/mol. This provides an opportunity for frequent proton-sharing events and proton transfer. In addition, the O-H${}^{BP}$ distance is shifted towards larger values of the bond suggesting that the intramolecular hydrogen bond is stronger than that obtained from the gas-phase results. The 2D free-energy surfaces (Figure 6) contain the donor-acceptor distance as the second coordinate. The results obtained for the gas phase showed that the O...N distance ranges between 2.35 Å–2.85 Å while the O-H${}^{BP}$ covers 0.9 Å–1.15 Å. The free-energy minimum was found between 0.99 Å–1.01 Å. The second free-energy minimum was not detected. The 2D free-energy surface obtained as a result of solid-state CPMD located two energy minima. The deeper one ranges 1.01 Å–1.08 Å and it is located on the proton-donor side. The second one is shallow, and it was found between 1.5 Å–1.75 Å of the O-H${}^{BP}$ distance. The O...N interatomic distance range was found shorter compared to the gas phase and it covers the 2.35 Å–2.78 Å range. An essential feature is that the path linking the minima travels from the donor-side minimum down the O...N distance, and then—after crossing the barrier—goes towards larger O...N distances. This modulation is a well-known feature of the strong, easily modifiable hydrogen bonds, but in the case of HBQ it is also directly linked to the experimentally observed coupling between the O-H stretching mode and the low-wavenumber structural modes involved in O...N distance changes [41]. Summarizing the simulations of HBQ molecule, the CPMD approach showed strong mobility of the bridged proton and the O-H${}^{BP}$ distance ranges between 0.9 Å–1.9 Å. The 2D free-energy surfaces obtained for it showed that the environmental effects strongly modulate the features of the intramolecular hydrogen bond.

In Figure S4 and Figure 6 the 1D and 2D Pmf results for the benzo[h]quinoline-2-methylresorcinol dimer are shown. The 1D Pmf profile obtained for the complex simulated in the gas phase indicated one free-energy minimum with the O-H distance of ca. 0.98 Å. Again, this result agrees with the structural study findings for the O-H...N${}^{a}$ intermolecular hydrogen bond in the dimer. In the solid state there is one deep free-energy minimum with O-H distance of ca. 1.01 Å and the second shallow minimum covering range ca. 1.28 Å–1.42 Å with the energy barrier equal 4.1 kcal/mol. The 2D free-energy profile for the gas-phase results showed that the O...N${}^{a}$ distance covers the range between 2.4 Å–3.4 Å. The O-H distance ranges between 0.9 Å–1.1 Å with the deeper part of the free-energy minimum between 0.98 Å–1.02 Å. An opposite situation was observed for the results of the solid-state simulations. The O...N${}^{a}$ distance was significantly shortened (it ranges between 2.4 Å–3.0 Å). The O-H distance was found between 0.9 Å–1.3 Å. The second minimum was also detected by the 2D surface. The Pmf results agree with our structure study where short contacts were noticed, see Figure 3.

**Figure 7:**
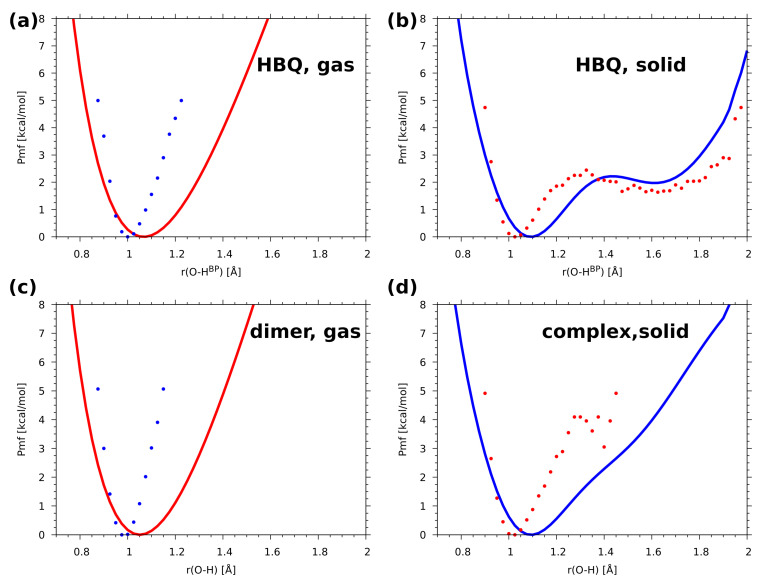
Comparison of the potential of mean force (Pmf) from the CPMD trajectory with classical nuclei (shown with circles) and with inclusion of a posteriori quantum corrections (drawn with lines) for proton motion in the investigated O-H...N hydrogen bridges (**a**): HBQ in the gas phase, (**b**): HBQ in the crystal, (**c**): the benzo[h]quinoline-2-methylresorcinol complex in the gas phase, (**d**): the complex in the crystal).

The incorporation of quantum effects into the Pmf (see Figure 7) leads to a rather significant broadening of the area accessible to the proton. Comparison of the classical 1D Pmf (Figure S4) with its a posteriori quantum-corrected analogue in Figure 7 shows that the gas-phase systems (HBQ as well as the dimer) do not exhibit secondary minima at the donor side, but the anharmonicity is much more pronounced in the quantum-corrected regime. A much more interesting Pmf profile is observed in the solid state for both compounds. In these cases, the anharmonicity is transformed into straightforward presence of the secondary, acceptor side, minimum for the HBQ, easily accessible with a small barrier of 2.1 kcal/mol, but also very easy to escape and revert to the donor-side localization of the proton. In case of the investigated heterodimer in the cocrystal, the secondary minimum is only very slightly marked by an inflexion point at ca. 1.55 Å. The quantum effects also move the location of the O-H distance minima towards larger value, 1.1 Å for the solid state and 1.05 Å in the gas phase. This is a significant increase from the classical Pmf locations of the minima (see Figure S4), related to the possibility of the nuclear wavefunction to penetrate regions of the potential energy space less accessible to the classical particle. The anharmonicity (asymmetry of the potential energy curve) makes this effect stronger towards the acceptor side. This also influences the observable position of the proton, which was demonstrated recently by Stare et al. [45]. In the current systems, the impact of the quantization of nuclear motions has led to the following oxygen donor—bridge proton distances: 1.0609 Å and 1.1912 Å in the gas-phase and solid-state HBQ simulations respectively, and further, 1.1520 Å and 1.1744 Å in the corresponding gas-phase and solid-state benzo[h]quinoline-2-methylresorcinol simulations. Comparison of these values with extended discussion of the metric parameters (see Tables S1 and S2 and the associated text in the Supplementary Material) shows the importance of the nuclear quantization for light nuclei in anharmonic potentials.

The explanation of the diversity of the molecular features in the studied systems, demonstrated in the previous sections, requires more thorough insight into the electronic structure of the two molecular assemblies. This insight will be provided in the next two sections.

### 2.3. Constrained Density Functional Theory (CDFT) Electronic Structure Analysis of Benzo[h]Quinoline-2-methylresorcinol Dimer and Trimers

The CDFT analysis was carried out for dimer and trimers derived from benzo[h]quinoline-2-methylresorcinol. Unfortunately, a single molecule cannot be investigated with this method without a significant amount of arbitrarity, as CDFT method assumes the charge transfer between the system components. There is no clear boundary within the 10-hydroxybenzo[h]quinoline (HBQ), which can be used to define the donor and acceptor parts of a given molecule, nor the amount of charge that needs to be transferred. For this reason we did not carry out the analysis of the intramolecular hydrogen bond in HBQ.

As far as the complexes with 2-methylresorcinol are concerned, we defined the donor and acceptor boundary along the intermolecular H-bond between the 2-methylresorcinol and benzo[h]quinoline. The charge transfer between the molecules forming the complex was identified in our previous work [30] to be caused by the electronic excitation. The relaxation of the complex was determined to be the radiationless one, occurring via the conical intersection of S0 and S1 states. The geometry of the intersection involves the proton from the hydroxyl group of the 2-methylresorcinol being transferred to the N atom of benzo[h]quinoline.

Within this part of the present work, we aimed to investigate the possibility of an electron-driven proton transfer (EDPT) between the molecules forming the complex. Table 1 shows the charge transfer energies calculated by means of the CDFT method, by imposing the constraint on the charge on the benzo[h]quinoline molecule (Table S3 in the Supplementary Material contains additional energy values for partial charge transfer scenarios). The investigated systems consisted also of one or two 2-methylresorcinol molecules in different configurations (see Figure 2). These, however, were not constrained, and the areas of charge depletion were subject of optimization. It can be noticed that the values of the charge transfer energies are of significant height, and vary between 5 and 6 eV. This implies that the EDPT is not thermodynamically favored, and this mechanism should not be considered to be plausible.

These values, however, do not match exactly the 3.4 eV value calculated using TD-DFT method in our previous study [30]. This discrepancy can be a result of many factors. First, the TD-DFT method and CDFT rely on different physical principles, with the latter employing an external potential to constrain the electron density on the particular atoms. This constraint is not present in the TD-DFT method, and as such, the complete separation of the charge between two molecules cannot be enforced. In addition, the calculations for the needs of this work were carried out in the periodic system, in which a dipole moment induced due to the charge transfer might affect the electronic structure and lead to the excessive height of the $E_{CT}$.

**Figure 8:**
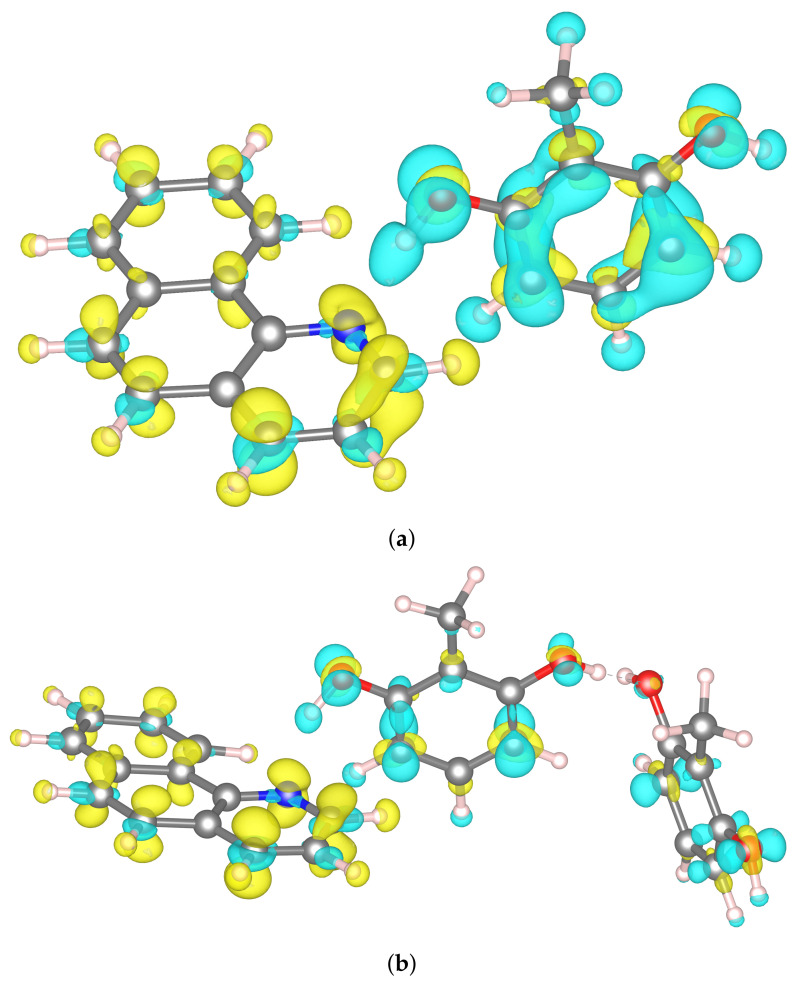

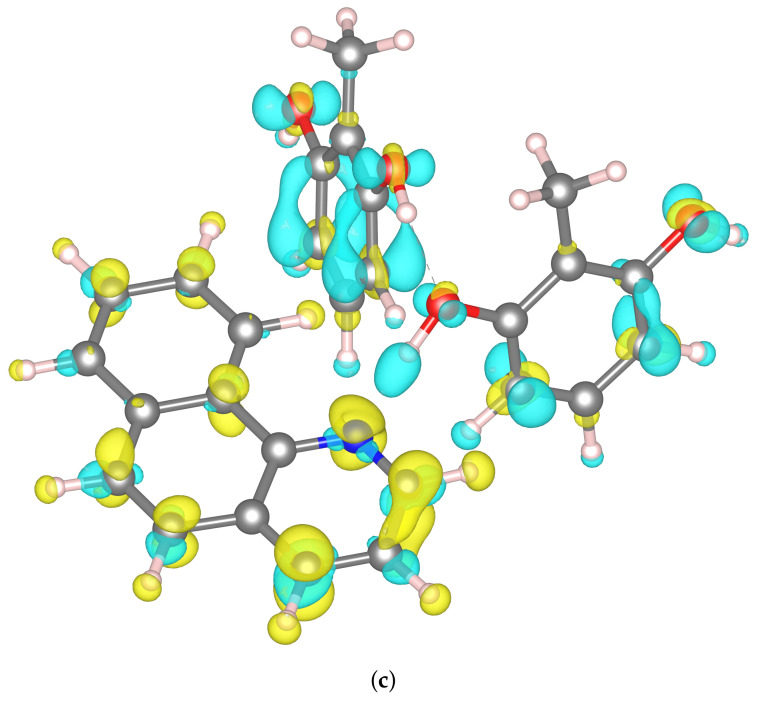
The differences in charge density calculated for the constrained electron transfer to the benzo[h]quinoline-2-methylresorcinol complexes: (**a**) dimer, (**b**) trimer 1, and (**c**) trimer 2. The yellow and blue areas represent the areas of the increased and decreased charge density, respectively. The isosurfaces are drawn for the value of 0.005.

Regardless of that, the qualitative results for the charge transfer in the dimer system are consistent. It can be noticed that the charge density difference is equivalent to the population of the π orbitals in benzo[h]quinoline (see Figure 8a). The charge depletion is observed on the π orbitals of the 2-methylresorcinol, what resembles the π→π* transition. Additionally, some repolarization of the charge density can be observed within each of the molecules forming the complex, which is represented by the electron density accumulation on the 2-methylresorcinol, which is an electron donor, and the electron density depletion on the benzo[h]quinoline molecule.

Similar observations could be made for the charge transfers in the trimers, in which 2 molecules of 2-methylresorcinol are present in the complex. Only minor differences were observed in the charge density distribution on the benzo[h]quinoline molecule. This behavior is expected, as in all cases one electron is transferred to the lowest-lying π orbital of the benzo[h]quinoline. In addition, in both trimers both 2-methylresorcinol molecules are the electron donors, even though no constraint was applied to any of them. Regardless of the relative orientation of 2-methylresorcinol molecules, the charge transfer energies are similar and they are lower than in the case of the dimer system. This can be explained by only partial draining of the electron density from both these molecules compared to the draining of a whole electron in the dimer system.

An interesting aspect of the CDFT analysis, is the charge depletion at the hydrogen atom forming the hydrogen bridge. This is visible in all three investigated systems as the blue area surrounding the hydrogen atom. This effect seems contradictory to the classical description of the hydrogen bond, in which the interaction is mainly of electrostatic nature, and the proton (understood as a point positive charge) is coordinated to the electron pairs at the oxygen and nitrogen atoms. This classical description, however, seems arbitrary at the point where the division line is drawn in between the molecules described above. In our case, we defined the 2-methylresorcinol molecule—including the H forming the H-bond—as the charge donor. This description seems oversimplified, and fails to fully describe the quantum effects of the system consisting of three centers, O-H...N. In order to shed more light on this effect, we have performed the analysis of the hydrogen bonding using Electron Localization Function.

### 2.4. Electron Localization Function (ELF) Topological Analysis of the Hydrogen Bonding Based on CPMD Trajectory

The results of the CDFT study suggest that it is worthwhile to investigate the electron density distribution in the diverse cases of hydrogen bonding encountered in the current study, namely the following questions should be answered: what are the populations of the electronic moieties contributing to the hydrogen bonds? How much charge is transferred or relocated when the proton position fluctuates in the molecular dynamics simulation? A method of answering these questions could be topological analysis of molecular fields related to the electron density, in particular Electron Localization Function (ELF).

Characterization of the hydrogen bonds based on ELF topological analysis was found useful in categorizing the weak, middle-strong, and strong cases of hydrogen bonding [46,47]. The distinction is based on the scheme of decomposition of the molecular space into particular sub-systems when the ELF isosurface value is changed. If the first separation splits the system into two separate molecules, the bonding is weak; if the first split yields a large valence space with embedded donor and acceptor moieties—the complex is of middle strength. The case of strong hydrogen bonding is similar, but the proton manifests itself as a separate basin, instead of being incorporated into the valence shell of an O-H or N-H bond. This is the most interesting case, and our studies showed its occurrence in some systems—see Table 2.

## 3. Computational Methods and Procedures

### 3.1. Car–Parrinello Molecular Dynamics in the Gas and Crystalline Phases

Car–Parrinello molecular dynamics (CPMD) [34] simulations were performed in the gas and the crystalline phases for 10-hydroxybenzo[h]quinoline (HBQ) and benzo[h] quinoline-2-methylresorcinol. The models for CPMD simulations were prepared based on crystal structures (CCDC entries: 1306650 [8] and 1060876 [9]) and they are presented in Figure 1 and Figure 2 as well as in Figure S1 (unit cells used during the solid states runs). The energy minimization was performed in both phases to prepare initial conditions for further CPMD runs. The prepared models for gas-phase simulations were placed to cubic cells with a = 13 Å for HBQ, a = 19 Å for the benzo[h]quinoline-2-methylresorcinol dimer, and a = 24 Å, a = 19 Å for the heterotrimers, respectively. The geometry optimization for the isolated systems was carried out with the center molecule option and using the initial Hessian matrix proposed by Schlegel [48]. The crystal of HBQ is orthorhombic with a = 4.6530 Å, b = 15.1910 Å, c = 26.902, Å and α. β, γ equal $90^{\circ }$ respectively and with Z = 8. The crystal of benzo[h]quinoline-2-methylresorcinol is monoclinic with a = 7.842 Å, b = 21.894 Å, c = 9.260 Å, α = $90^{\circ }$, β = $106.28^{\circ }$ and γ = $90^{\circ }$ with Z = 4. The crystalline phase energy minimization was performed with Γ point approximation (i.e., using Bloch eigenfunctions with 0 reciprocal vector k to represent the periodicity of the crystal) [49] and periodic boundary conditions (PBCs) and with real-space electrostatic summations for the six nearest neighbors in each direction (TESR = 6). The exchange-correlation functional PBE [50,51] coupled with planewave basis set and Troullier-Martins [52] pseudopotentials were employed. A kinetic energy cutoff for the planewave basis set of 100 Ry was used for both compounds and in both simulated phases. The fictitious electron mass parameter was equal to 400 a.u. and the time-step was set to 2 a.u. The empirical van der Waals corrections proposed by Grimme [53] were added to reproduce weak interactions. Next, the CPMD simulations were performed using the general set up described above. Hockney’s scheme [54] was used to remove interactions with periodic images of the cubic cell. Additionally, the translational and rotational movements were removed during the CPMD runs in the gas phase. The simulations were performed at 297 K. In order to control the assigned condition a Nosé–Hoover thermostat chain was applied [55,56]. Initially, the investigated systems were equilibrated, and the data (production run of the CPMD) were collected for ca. 15 ps. The obtained trajectories served for further post-processing data analyses. The time evolution of interatomic distances of atoms involved in the intra- and intermolecular hydrogen bonds formation. The spectroscopic features of the investigated compounds were obtained by Fourier transformation of atomic velocity values into vibrational signatures.

### 3.2. A Posteriori Inclusion of Quantum Effects of Nuclear Motion of the O-H Stretching

The O-H stretching envelope [31] method was applied to incorporate quantum effects of nuclear motion. The procedure is as follows: first, the CPMD trajectory was sampled with constant increment equal to 0.72 ps (15,000 time steps) to obtain snapshots (22 for the gas and crystalline phases for HBQ as well as for benzo[h]quinoline-2-methylresorcinol complex). The obtained snapshots were used as starting points to perform rigid scan calculations of the bridged proton reaction path. A set of proton potential functions was achieved. Depending on the O...N and O...O interatomic distances, a set of 16 to 18 proton positions were found along a circular arc formed between the donor and acceptor atoms in the hydrogen bridge [57,58]. Then, the potential energy surface (PES) scans gave proton potential functions used further to solve the vibrational Schrödinger equations [59]. Finally, the O-H stretching envelope was constructed based on the vibrational eigenvalues by superimposing a set of Gaussian functions with small half-width (100 ${\mathrm{cm}}^{-1}$) centered at the respective eigenvalues.

### 3.3. One-Dimensional (1D) and Two-Dimensional (2D) Potential of Mean Force (Pmf)

1D and 2D Pmf for proton motion in the O-H...N hydrogen bridges were calculated to reproduce free-energy profiles (along the postulated reaction coordinate) [60]. Two approaches were applied for this purpose:

(i) calculations of the Pmf based on CPMD trajectory, where atomic nuclei are treated classically. In this case, the whole trajectory is used to derive the probability density distribution ρ(r) (histogram with 0.025 Å bin width) for the O-H bond length internal coordinate. Then, the potential of mean force (corresponding to the free-energy landscape) is obtained directly from the formula $Pmf\left(r\right)=-k_{B}Tln\rho \left(r\right)$, where $k_{B},T$ are the Boltzmann constant, and simulation temperature respectively.

(ii) calculations of the Pmf using CPMD trajectory snapshots where the quantum effects were incorporated a posteriori. More details about the second approach are given below or in Refs. [61,62]. Basically, the snapshots extracted and processed in the section on the a posteriori quantization were used (22 snapshots from each calculation type). The 1D Pmf with quantum effects inclusion was calculated for the gas phase and crystalline phases. It was computed using eigenvalues and eigenfunctions being a result of the vibrational Schrödinger equation [59] solution based on the snapshots taken from the CPMD runs. The eigenfunctions provide probability distributions for each snapshot, which are then averaged over the set of snapshots from the CPMD trajectories. Subsequently, the statistical average is used to obtain the Pmf. The calculations of Pmf were performed based on the vibrational ground-state wavefunctions and the two or three least excited vibrational states.

The ab initio molecular dynamics simulations were performed with the CPMD 3.17.1 program [63]. The post-processing and data visualization were carried out using the VMD 1.9.3 [64], the Mercury 3.1 [65] and Gnuplot [66] programs as well as home-made scripts (for Fourier transform autocorrelation function of atomic velocity, calculation of histograms for 1D and 2D Pmf).

### 3.4. Constrained Density Functional Theory (CDFT) Method

The Constrained DFT (CDFT) calculations were carried out for dimer and trimers of benzo[h]quinoline-2-methylresorcinol complex with solvent reaction field using water as a solvent. The initial geometries were taken from the CPMD geometry optimization results. The charge density changes of the O-H...N proton reaction paths were examined. The CDFT simulations were carried out using the implementation of Holmberg and Laasonen [67,68] in the CP2K code version 8.0 [69]. The method is based on an external constraint potential acting on the electron density of the ground state and modifying its distribution according to the given parameters [67]. Imposing a charge constraint allows the movement of electrons within the system in a controlled manner to represent the charge transfer state. A comparison of the energy of such state with the energy of a ground state obtained from conventional DFT yields the energies of a charge transfer—$E_{CT}$. In our simulations, we used the PBE functional [50,51] with the Gaussian-planewave approach. We used localized, atom-centered DZVP-MOLOPT-SR-GTH basis set [70], and the planewave basis with the 600 Ry cutoff as the auxiliary basis. The charge was constrained with weight function in form of Becke space partitioning scheme [71]. To impose the constraint on a particular molecule, we set the target parameter, increasing or decreasing charge value of the component by a unit charge. The initial constraint strength was set to −0.1 and was subjected to optimization within the CDFT procedure. The SCF convergence criterion was set to 1 × 10^−6^ and for the outer loop SCF to 1 × 10^−3^ *e*. The element specific cutoffs were used with the values of 2.28 Å and 2.65 Å for hydrogen atoms and all elements heavier than hydrogen, respectively, along with the paper of Holmberg and Laasonen [67]. Additionally, a Gaussian cavity confinement shaped with van der Waals radii were used with a density threshold for cavity creation set to 1 × 10^−7^. The solvent effects were included by means of the self-consistent continuous solvation method of Andreussi et al. [72]. The dielectric constant of the solvent was set to 78.36 and the surface tension to 0.0. The visualization of the charge density differences was achieved with the VESTA code, version 3.5.7 [73].

### 3.5. Electron Localization Function (ELF)

The Electron Localization Function (ELF) topological analysis was applied to study the molecular properties of the intra- and intermolecular hydrogen bonds in the light of the bridged proton dynamics based on CPMD trajectories [38]. In the current study, the ELF analysis was performed for the structures extracted from the gas phase and the crystal simulations for the O-H...N hydrogen bonds [46]. The CPMD trajectories were sampled with constant simulation time intervals and a set of 22 snapshots was obtained from each CPMD run. Then, for the snapshots indicating various bridged proton positions (e.g., proton at the donor side, a proton in the middle of the hydrogen bridge, and proton at the acceptor-atom side—if the proton-transfer phenomenon was registered), wavefunctions were computed using the Gaussian 16 Rev. C.01 suite of programs [74]. Furthermore, the ELF and electron density were calculated for each structure using a fine grid of 0.05 $a_{0}$. The topological basins of ELF were then determined, and electron density was integrated within each ELF basin. A similar procedure was applied in one of our previous studies [75]. The calculations were performed at the DFT level of theory [76,77] with the Perdew, Burke, and Ernzerhof functional denoted as PBE [50,51] and basis set def2-TZVP [78,79]. The computations were carried out with the DGrid 5.1. package [80].

## 4. Conclusions

Car–Parrinello molecular dynamics (CPMD) and Constrained Density Functional Theory (CDFT) simulations were carried out for 10-hydroxybenzo[h]quinoline (HBQ) and benzo[h]quinoline-2-methylresorcinol complex. The computations were performed in vacuo, in water (using continuous solvation model) and in the crystalline phase. The O-H...N and O-H...O intra- and intermolecular hydrogen bonds features were studied. The interactions have been considered to be the strongest among non-covalent forces. Thus, the time evolution of metric parameters of the hydrogen bonds was analyzed as well as the vibrational properties. Additionally, the electronic structure changes depending on the bridged proton position were examined based on CDFT and Electron Localization Function (ELF) methods. The CPMD results for the gas-phase simulations showed that the bridged protons are localized on the donor side in HBQ molecule and dimer and trimers of benzo[h]quinoline-2-methylresorcinol complex. However, the proton-transfer phenomena were observed for the HBQ and proton-sharing events for both investigated systems in the crystalline phase. The computed power spectra of atomic velocity provided spectroscopic signatures for the investigated systems, and they agree with the metric findings concerning the bridged protons positions and strengths of the O-H...N and O-H...O hydrogen bonds. In addition, they are in good agreement with the experimental data available. Concluding, the spectroscopic properties were well reproduced by CPMD approach for HBQ and benzo[h]quinoline-2-methylresorcinol in the gas phase and in the solid state. The incorporation of quantum effects to the O-H stretching and Pmf yielded significant shift of the $\nu_{OH}$ stretching mode towards lower wavenumbers, and allowed the proton to sample the acceptor space more efficiently. These facts are direct consequences of the increase in the anharmonicity of the potential energy well for the proton motion. An application of CDFT method provided an insight in the charge density differences of the systems prior to and after the electron transfer. These results brought to light the importance of the quantum effects involved in the charge transfer—contrary to a simplistic, classical description, the data suggest that the three-center interactions need to be considered to account for the charge density difference. Finally, the ELF study showed that only the solid-state simulation of HBQ contains complete proton-transfer events. The second strongest is the gas-phase HBQ molecule, exhibiting numerous proton-in-the-middle instances. Among the studied O-H...N^a^ bridges, the intermolecular bridge in the benzo[h]quinoline-2-methylresorcinol complex was the weakest according to the topological analysis, which correctly identified the modes of behavior in the studied systems. The sensitivity of the hydrogen bonding in the studied benzo[h]quinolines is thus demonstrated. We have revealed the ease of modification of the interactions in the benzo[h]quinoline derivatives by the crystal environment, quantum corrections and electron density redistribution.

## Figures and Tables

**Table 1:** Charge transfer energies ($E_{CT}$) calculated for the benzo[h]quinoline-2-methylresorcinol complexes shown in Figure 2.

System	$E_{\mathrm{CT}}$ [eV]
dimer	6.12
trimer 1	5.03
trimer 2	5.13

**Table 2:** Populations (minimum–maximum) of the ELF basins related to the O-H...N hydrogen bridges in HBQ (gas phase and solid) and the benzo[h]quinoline-2-methylresorcinol complex (gas phase). The first row for each of the three bridge structures show the number of reported cases from among 22 snapshots. The possible structures are, from top to bottom respectively, the proton at the donor side, a proton in the middle, and proton at the acceptor side.

Parameter	HBQ, Gas Phase	HBQ, Solid	Complex, Gas Phase
n(O-H^BP^...N)	20	17	22
V(O-H^BP^)	1.764–1.838	1.759–1.842	1.647–1.835
V(N)	2.684–2.867	2.755–2.854	2.686–2.820
n(O...H^BP^...N)	2	2	0
V(O)	1.263–1.355	1.235–1.249	–
V(H^BP^)	0.486–0.554	0.529–0.547	–
V(N)	2.664–2.800	2.621–2.859	–
n(O...H^BP^-N)	0	3	0
V(O)	–	2.557–2.646	–
V(H^BP^-N)	–	2.453–2.908	–

## Data Availability

The data presented in this study are available in the article and in the associated Supplementary Material.

## Supplementary Materials

The following are available online at https://www.mdpi.com/article/10.3390/ijms22105220/s1, Figure S1: Unit cells of 10-hydroxybenzo[h] quinoline (HBQ) [1] and benzo[h] quinoline-2-methylresorcinol [2] used for CPMD simulations in the crystalline phase, Table S1: Selected structural parameters related to the intramolecular hydrogen bridge and quasi-ring in 10-hydroxybenzo[h]quinoline (HBQ). Comparison of experimental (X-ray) [1] and computed (CPMD) results, Figure S2: Unit cell of benzo[h]quinoline-2-methylresorcinol cocrystal [2] with molecules forming the benzo[h]quinoline-2-methylresorcinol dimer, as well as the two investigated trimers, Table S2: Selected structural parameters related to the intermolecular hydrogen bridges in benzo[h]quinoline-2-methylresorcinol complexes, Figure S3: Time evolution of the metric parameters of the intermolecular O-H...O^b^ hydrogen bridges in the investigated benzo[h]quinoline-2-methylresorcinol complex in the solid state, Figure S4: Potential of mean force (Pmf) for proton motion in the O-H...N hydrogen bridges computed from the CPMD trajectories obtained as results of gas phase and solid-state simulations, Table S3: Charge transfer energies (E${}_{CT}$) for the partial charge transfer between the system components.

## Abbreviations

The following abbreviations are used in this manuscript:

CDFT	Constrained Density Functional Theory
CPMD	Car–Parrinello Molecular Dynamics
DFT	Density Functional Theory
ELF	Electron Localization Function
HB	Hydrogen bond
HBQ	10-hydroxybenzo[h]quinoline
Pmf	Potential of mean force
