# Supplementary material for: Sensitivity of Intra- and Intermolecular Interactions of Benzo[h]quinoline from Car–Parrinello Molecular Dynamics and Electronic Structure Inspection
Source: Int J Mol Sci. 2021 May 14;22(10):5220. doi: 10.3390/ijms22105220 (PMC8156133; doi:10.3390/ijms22105220)
Supplement: Supplementary file 1 [file ijms-22-05220-s001.zip › ijms-1205149-supplementary.pdf]

SUPPLEMENTARY INFORMATION

Sensitivity of intra- and intermolecular interactions of benzo[h]quinoline from Car-Parrinello molecular dynamics and electronic structure inspection

Jarosław J. Panek†, Joanna Zasada‡, Bartłomiej M. Szyja‡, Beata Kizior‡ and Aneta Jezierska†*

† University of Wrocław, Faculty of Chemistry, F. Joliot-Curie 14, 50-383 Wrocław, Poland

‡ Wroclaw University of Science and Technology, Department of Fuels Chemistry and Technology, ul. Gdańska 7/9, 50-344Wroclaw, Poland

*Corresponding author: Aneta Jezierska, aneta.jezierska@chem.uni.wroc.pl

phone. +48 71 3757 224; fax: +48 71 3282 348

Table of contents:

1. Car-Parrinello metric parameters discussion of 10-hydroxybenzo[h]quinoline (HBQ) and benzo[h]quinoline-2-methylresorcinol complex. …………………..………………. **3**
2. **Figure S1.** Unit cells of 10-hydroxybenzo[h]quinoline (HBQ) [1] (left) and benzo[h]quinoline-2-methylresorcinol [2] (right) used for CPMD simulations in the crystalline phase. ……………………………………………………………….. **7**
3. **Table S1.** Selected structural parameters related to the intramolecular hydrogen bridge and *quasi*-ring in 10-hydroxybenzo[h]quinoline (HBQ). Comparison of experimental (X-ray) [1] and computed (CPMD) results. Metric parameters are given in Å and degrees. For the CPMD the average±standard deviation was estimated. …….………**8**
4. **Figure S2.** Unit cell of benzo[h]quinoline-2-methylresorcinol cocrystal [2] with molecules forming the benzo[h]quinoline-2-methylresorcinol dimer, as well as the two investigated trimers (identified by color markings). Intermolecular hydrogen bonds are indicated with dotted lines. ………………………………………………………….. **9**
5. **Table S2.** Selected structural parameters related to the intermolecular hydrogen bridges in benzo[h]quinoline-2-methylresorcinol complexes (gas phase dimer and two trimers - see Figure 2 and cocrystal (Figure S2). Comparison of experimental (X-ray) [2] and computed (CPMD simulations) results. Metric parameters are given in Å and degrees. For the CPMD the average±standard deviation was estimated. ……..…… **10**

6. **Figure S3.** Time evolution of the metric parameters of the intermolecular O-H...O hydrogen bridges in the investigated benzo[h]quinoline-2-methylresorcinol complex in the solid state. Left: the O2-H2…O^b bridge, in which the central 2-methylresorcinol molecule acts as a hydrogen bond donor; right – the $O3^c$-$H3^c$…O bridge, in which the central molecule is a hydrogen bond acceptor. Color coding of the graphs: red - d(O...O), green - r(O-H), blue: r(H...O). The O^b atom is generated from the O atom in the crystal structure by symmetry operation (½+x, ½–y, ½+z), while the $O3^c$-$H3^c$ atoms are generated from the O2 atom in the crystal structure by symmetry operation (–½+x, ½–y, –½+z). ………………………………………………………….….. **11**
7. **Figure S4.** Potential of mean force (Pmf) for proton motion in the O-H…N hydrogen bridges computed from the CPMD trajectories obtained as results of gas phase and solid state simulations. Two sections are provided: Pmf calculated directly from the CPMD trajectories (classical nuclei), and Pmf calculated with *a posteriori* quantum corrections obtained during the "snapshot-envelope" calculations. Upper graphs in each section: results for HBQ; lower graphs: benzo[h]quinoline-2-methylresorcinol complex. ……………………………………………………………………….…. **12**
8. **Table S3.** Charge transfer energies (E_{CT}) for the partial charge transfer between the system components. The values are given in eV. ………………………...……… **13**

Car-Parrinello metric parameters discussion of 10-hydroxybenzo[h]quinoline (HBQ) and benzo[h]quinoline-2-methylresorcinol complex

10-hydroxybenzo[h]quinoline (HBQ)

Two independent molecules of HBQ are present in the unique part of the unit cell, and the O...N interatomic distance is equal 2.562 Å or 2.574 Å according to the experimental measurements [1]. This shows the extent of modification of intramolecular hydrogen bond properties just by the crystal environment. The distance obtained as a CPMD result in the gas phase is elongated comparing to the X-ray findings, see Table S1 (and for clarity Figure 1 in the manuscript body). The O-H^{BP} experimental value suggests that the proton is located in the middle of the hydrogen bridge (1.337 Å or 1.361 Å). The average value of the O-H^{BP} bond is equal 1.022±0.037 Å, which indicates that the proton is located on the donor side. The hydrogen H^{BP}...N bond length is 1.411 Å or 1.454 Å according to the X-ray measurements, but the computed average value is 1.670±0.115 Å. Therefore, the discrepancies between the experimental and gas phase computed data are noticeable (ca. 0.2 Å), underlining the difference between the solid structure and the gas phase. Concerning the valence angle $OH^{BP}N$, the atoms involved in the intramolecular hydrogen bond formation exhibited flexibility during the CPMD run, therefore the obtained value is larger than the experimental. The O-C1 and C3-N bond lengths average values are in good agreement with experimental data, however, they are slightly shorter. The C1-C2 and C2-C3 bond lengths average values comparison with the X-ray data showed that they were reproduced correctly, and - most important - the pattern of elongations and shortenings, corresponds to the modifications in aromaticity, is also reproduced. As it has already been mentioned, in the crystal of HBQ, there are two symmetry-independent molecules. Both geometries are similar, but not identical [1] as it is shown in Table S1 the standard deviations of the structural parameters in the CPMD runs are much larger than these differences. The crystalline phase CPMD simulation results are in good agreement with the X-ray measurements. The computed O...N interatomic distance was obtained as slightly shorter. The O-H^{BP} bond value obtained as a result of the CPMD simulation suggests that the enol form of the HBQ molecule is preferable. The H^{BP}...N hydrogen bond computed average value is equal 1.496±0.218 Å and it is elongated comparing with the experiment (1.411 Å or 1.454 Å). The valence angle $OH^{BP}N$ computed value differs significantly from the experimental findings. It is an unexpected result - a better agreement could be expected, because the simulation of the unit cell took into account long and short-

term interactions. Therefore, the degrees of freedom of the molecules are limited by e.g. the neighbors or crystal field. Concerning the *quasi*-ring remaining bond lengths, the O-C1 and C3-N computed average bond lengths are shorter comparing to the experimental data. This could be associated with the fact that oxygen and nitrogen atoms of the bonds are involved in the intramolecular hydrogen bond and the bridge dynamics influenced the bond length as well as the presence of the neighboring molecules. The C1-C2 bond length average value is slightly elongated comparing with the reference data. However, the C2-C3 bond length obtained computationally corresponds perfectly with the experimental measurements. The difference in metric parameters of the molecules measured experimentally is ca. 0.01 Å. As it is reported in the Table S1, the computed standard deviation is ca. 0.07 Å, therefore, the CPMD approach would not be able to reproduce the slight difference in the geometry of the molecules.

Benzo[h]quinoline-2-methylresorcinol complex

The metric parameters of benzo[h]quinoline-2-methylresorcinol complex are listed in Table S2 for gas phase and solid state CPMD simulations (for clarity see Figure 2 in the manuscript body). The gas phase simulation results for the dimer (benzo[h]quinoline-2-methylresorcinol) reproduced the O...N^a distance with a slight elongation (0.08 Å) comparing to the X-ray experimental data [2]. However, as it is shown in Table S2 the experimental O-H bond was shortened, but the CPMD results provided us with the average distance of 1.002 Å, which is in agreement with literature reports. The average value of the hydrogen bond length was obtained shorter than the experimental one. The computed OHN^a valence angle value is in a very good agreement with experimental findings. In the crystalline phase simulation, the obtained O...N^a interatomic distance was shortened ca. 0.073 Å comparing to the X-ray measurements. The O-H bond length was reproduced correctly as well as the OHN^a valence angle. The H...N^a hydrogen bond was shortened noticeably, because the difference between computed and experimental value is 0.321 Å, but this results from experimental difficulties in locating the hydrogen-bonded proton (the distance of 0.795 Å is too short). The hydrogen-bonded protons bound to strongly electronegative atoms suffer most from the distortion of the electron cloud, hence the largest mismatch between the X-ray measurements and actual position of the nucleus. The trimers consist of one benzo[h]quinoline molecules and two molecules of 2-methyresorcinol, see Figure 2 in the manuscript body. The comparison of geometric and computed selected metric parameters for trimers 1 and 2 is shown in Table S2.

The trimer 1 in the gas phase corresponds to the case of two separated hydrogen bridges, formed respectively by two hydroxyl groups of the 2-methylresorcinol. The O...N^a bridge is well conserved with respect to the crystal - its elongation due to the lack of crystal field is only 0.07 Å, and the valence angle is also reproduced accurately. A sharp contrast is provided by the O2...O^b bridge, which undergoes very large structural fluctuations. The O...N^a distance is as much as 0.99 Å longer than in the experiment, and examination of the trajectory, as well as the time evolution of the parameters, showed that the second 2-methylresorcinol molecule is able to detach for a significant part of the simulation. This fact is also reflected in the standard deviation values of the second bridge parameters, and it strongly suggests that the O2-H2...O^b bridge is much weaker than its O-H...N^a counterpart. The crystalline phase simulation of trimer 1 reproduced the O2...O^b interatomic distance with a very good agreement. The O2-H2 bond length experimental value is shorter than that reported in the literature, however, the CPMD simulation results indicated that the bond is equal ca. 1 Å. The H2...O^b hydrogen bond average length is shorter comparing to the X-ray data (the difference is equal 0.24 Å). The same remark about the experimental O-H bond length being too short, already stated above, holds also here and explains the discrepancies between the X-ray diffractometry and CPMD simulation. The O2H2O^b valence angle computed differs ca. 6 degrees with respect to the experimental findings. The gas phase simulation of trimer 2 yields results differing significantly from those of trimer 1. The three molecules stay close together throughout the CPMD run, and the standard deviations of the distances are not large. The O...N^a bridge is strengthened and shortened in comparison to the dimer and trimer 1 simulations, and its length, 2.722 Å, is within experimental error of the X-ray result. This strengthening must be put down to the polarization of this bridge by the $O3^c$-$H3^c$ hydroxyl group. This interesting phenomenon play important role in formation of the structure of the investigated cocrystal [2]. With regard to the part of the crystal structure constituting the trimer 2, the $O3^c$...O interatomic distance obtained from the experiment is 2.750 Å and comparing with the computational average results it is ca. 0.046 Å larger. The $O3^c$-$H3^c$ bond length experimentally determined is shorter than that obtained computationally. The computed average value of the intermolecular hydrogen bond is 1.731±0.136 and it is shorter than the experimental one. Some differences were also observed concerning the $O3^cH3^c$O valence angle - the experimental reported value is equal 171.31 degrees and it is larger by ca. 6 degrees comparing to the CPMD results. The results gathered in Table S2 show an interesting feature of the solid state simulation for the benzo[h]quinoline-2-methylresorcinol cocrystal. Please note that the two trimers selected for the gas phase simulations, consisting of

one benzo[h]quinoline moiety and two 2-methylresorcinol molecules, are derived from the crystal structure in which only one 2-methylresorcinol molecule is independent. Therefore, the X-ray data in the last two sections of Table S2 are identical by virtue of crystal symmetry. The CPMD simulation uses full unit cell and assumes no symmetry, but still the last column of this Table yields identical results (within 0.002 Å) for the corresponding distances. This is a good indication of the convergence of the CPMD results, even if the particular details of the metric parameters time evolution differ between the two bridges (see Figure S3).

Comparing experimental solid state measurements with computed gas phase data, it is necessary to remember that the isolated molecule degrees of freedom are not limited by interactions present in the crystalline phase therefore some discrepancies could be noted. In addition, the X-ray measurements provided us only with probable bridged proton position. However, our CPMD simulations in the gas and solid states refilled the experimental data available as well as shed light onto proton dynamics in the hydrogen bridges of the studied systems. In general, the computationally obtained CPMD results are in good agreement with experimental X-ray data.

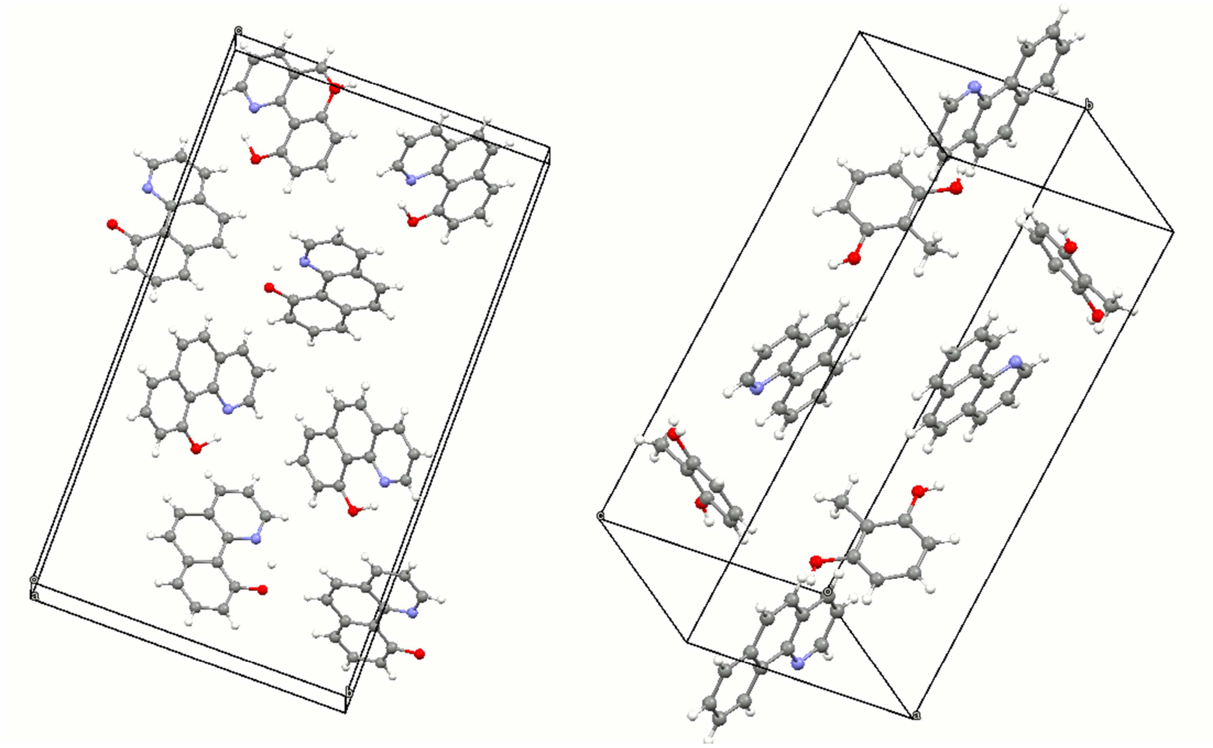

Figure S1. Unit cells of 10-hydroxybenzo[h]quinoline (HBQ) [1] (left) and benzo[h]quinoline-2-methylresorcinol [2] (right) used for CPMD simulations in the crystalline phase.

Table S1. Selected structural parameters related to the intramolecular hydrogen bridge and *quasi*-ring in 10-hydroxybenzo[h]quinoline (HBQ). Comparison of experimental (X-ray) [1] and computed (CPMD) results. Metric parameters are given in Å and degrees. For the CPMD the average±standard deviation was estimated.

Parameter	X-ray[a]	HBQ, gas phase	HBQ, crystal
**d(O…N)**	2.562, 2.574	2.592±0.081	2.555±0.079
**r(O-H^{BP})**	1.337, 1.361	1.022±0.037	1.152±0.221
**r(H^{BP}…N)**	1.411, 1.454	1.670±0.115	1.496±0.218
**<(OH^{BP}N)**	137.63, 132.17	148.51±5.66	149.57±6.20
**r(O-C1)**	1.368, 1.369	1.346±0.027	1.339±0.033
**r(C1-C2)**	1.421, 1.415	1.434±0.030	1.451±0.030
**r(C2-C3)**	1.440, 1.443	1.442±0.030	1.443±0.028
**r(C3-N)**	1.371, 1.368	1.364±0.028	1.356±0.027

[a]There are two independent molecules in the unique part of the unit cell.

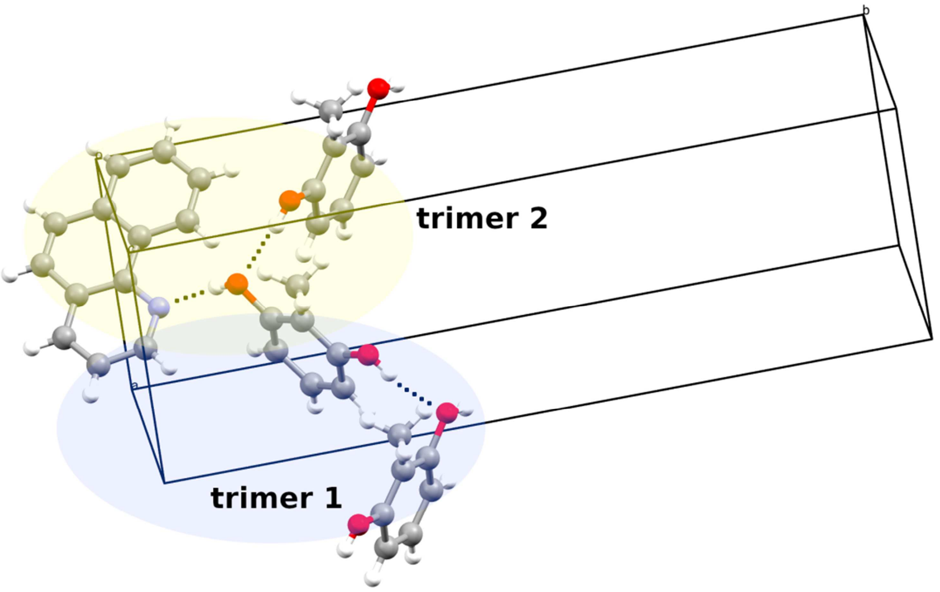

Figure S2. Unit cell of benzo[h]quinoline-2-methylresorcinol cocrystal [2] with molecules forming the benzo[h]quinoline-2-methylresorcinol dimer, as well as the two investigated trimers (identified by color markings). Intermolecular hydrogen bonds are indicated with dotted lines.

Table S2. Selected structural parameters related to the intermolecular hydrogen bridges in benzo[h]quinoline-2-methylresorcinol complexes (gas phase dimer and two trimers - see Figure 2 and cocrystal (Figure 2SI). Comparison of experimental (X-ray) [2] and computed (CPMD simulations) results. Metric parameters are given in Å and degrees. For the CPMD the average±standard deviation was estimated.

Parameter	X-ray	dimer	trimer 1	trimer 2	simulated cocrystal
**d(O…N[a])**	2.723	2.803±0.138	2.791±0.132	2.722±0.122	2.650±0.098
**r(O-H)**	0.795	1.002±0.030	1.002±0.030	1.020±0.036	1.044±0.049
**r(H…N[a])**	1.955	1.841±0.153	1.830±0.143	1.729±0.138	1.634±0.126
**<(OHN[a])**	162.39	161.90±8.85	161.25±8.48	165.09±6.97	164.13±7.04
**d(O2…O[b])**	2.750	-	3.741±0.728	-	2.705±0.118
**r(O2-H2)**	0.783	-	0.978±0.024	-	0.998±0.029
**r(H2…O[b])**	1.973	-	3.181±0.851	-	1.733±0.131
**<(O2HO[b])**	171.31	-	161.25±8.48	-	165.56±7.49
**d(O3[c]…O)**	2.750	-	-	2.847±0.180	2.704±0.123
**r(O3[c]-H3[c])**	0.783	-	-	0.988±0.026	0.998±0.029
**r(H3[c]…O)**	1.973	-	-	1.902±0.194	1.731±0.136
**<(O3[c]H3[c]O)**	171.31	-	-	161.48±9.77	165.79±7.46

[a] Symmetry code N(x, y, z) in the crystal structure;

[b] Symmetry code O(½+x, ½–y, ½+z) in the crystal structure;

[c] Symmetry code O2(–½+x, ½–y, –½+z) in the crystal structure.

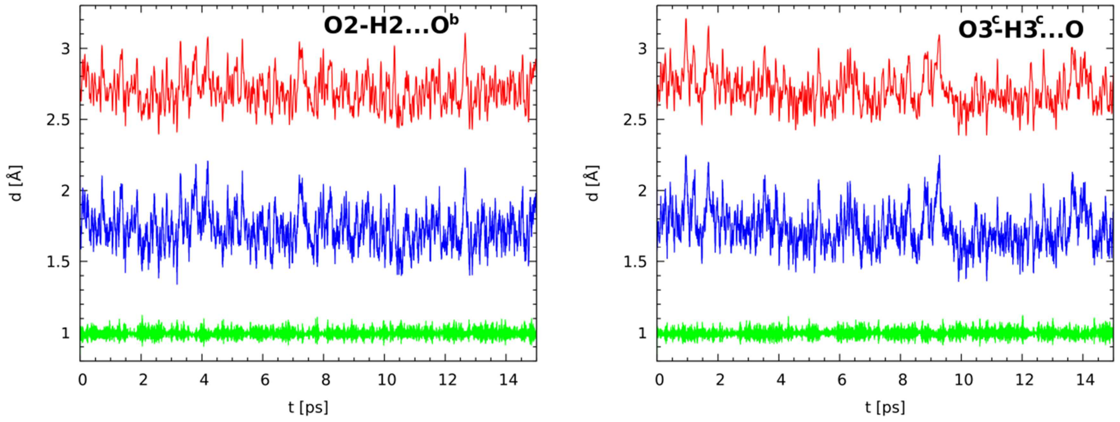

Figure S3. Time evolution of the metric parameters of the intermolecular O-H...O hydrogen bridges in the investigated benzo[h]quinoline-2-methylresorcinol complex in the solid state. Left: the O2-H2…O^b bridge, in which the central 2-methylresorcinol molecule acts as a hydrogen bond donor; right – the $O3^c$-$H3^c$ …O bridge, in which the central molecule is a hydrogen bond acceptor. Color coding of the graphs: red - d(O...O), green - r(O-H), blue: r(H...O). The O^b atom is generated from the O atom in the crystal structure by symmetry operation (½+x, ½–y, ½+z), while the $O3^c$-$H3^c$ atoms are generated from the O2 atom in the crystal structure by symmetry operation (–½+x, ½–y, –½+z).

Pmf - RESULTS FOR CLASSICAL NUCLEI

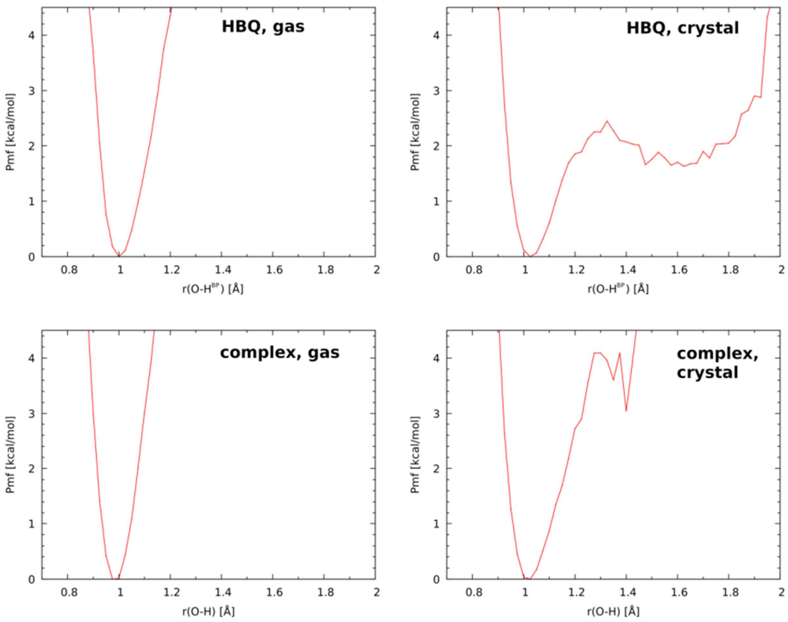

Pmf - RESULTS FOR QUANTUM NUCLEI (*a posteriori* correction)

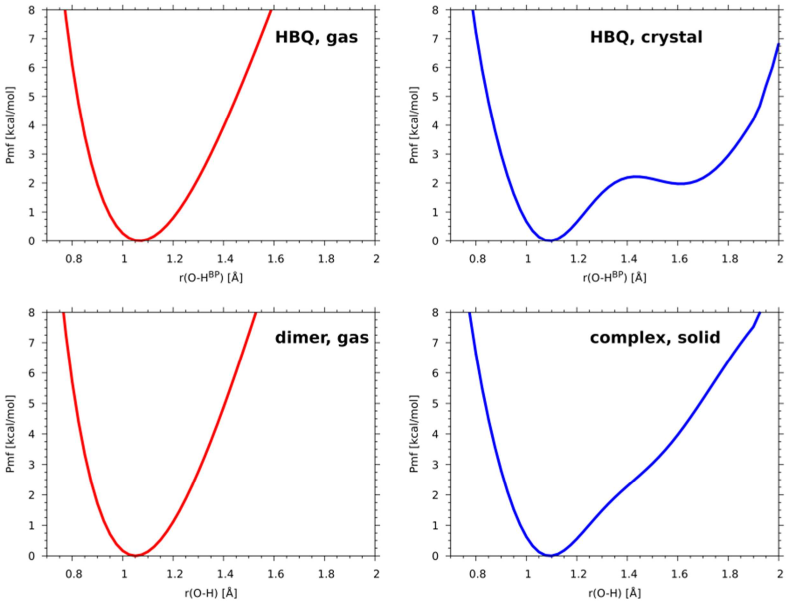

Figure S4. Potential of mean force (Pmf) for proton motion in the O-H…N hydrogen bridges computed from the CPMD trajectories obtained as results of gas phase and solid state simulations. Two sections are provided: Pmf calculated directly from the CPMD trajectories (classical nuclei), and Pmf calculated with *a posteriori* quantum corrections obtained during the "snapshot-envelope" calculations. Upper graphs in each section: results for HBQ; lower graphs: benzo[h]quinoline-2-methylresorcinol complex.

Table S3. Charge transfer energies (E_{CT}) for the partial charge transfer between the system components. The values are given in eV.

	**dimer**	**trimer 1**	**trimer 2**
**0.5e**	3.12	2.49	2.59
**0.6e**	3.66	2.95	3.04
**0.7e**	3.89	3.43	3.53
**0.8e**	4.47	3.94	4.04
**0.9e**	5.46	4.48	4.57
**1.0e**	6.12	5.03	5.13

References:

[1] Kubicki, M.; Borowiak, T. 10-Hydroxybenzo[h]quinoline. *Acta Cryst.* **1995**, *C51*, 1173-1175.

[2] Mir, N.A.; Dubey, R.; Tothadi, S.; Desiraju, G.R. Combinatorial crystal synthesis of ternary solids based on 2-methylresorcinol. *CrystEngComm*, **2015**, *17*, 7866-7869.
